# Genome-wide identification of Chiari malformation type I associated candidate genes and chromosomal variations

**DOI:** 10.3906/biy-2009-19

**Published:** 2020-12-14

**Authors:** Timuçin AVŞAR, Şeyma ÇALIŞ, Baran YILMAZ, Gülden DEMİRCİ OTLUOĞLU, Can HOLYAVKİN, Türker KILIÇ

**Affiliations:** 1 Department of Medical Biology, School of Medicine, Bahçeşehir University, İstanbul Turkey; 2 Neuroscience Program, Health Sciences Institute, Bahçeşehir University, İstanbul Turkey; 3 Neuroscience Laboratory, Health Sciences Institute, Bahçeşehir University, İstanbul Turkey; 4 Molecular Biology, Genetics, and Biotechnology Graduate Program, Graduate School of Science, Engineering, and Technology, İstanbul Technical University, İstanbul Turkey; 5 Department of Neurosurgery, School of Medicine, Bahçeşehir University, İstanbul Turkey

**Keywords:** Chiari type I malformation, neurogenetics, microarray analysis, molecular karyotyping

## Abstract

Chiari malformation type I (CMI) is a brain malformation that is characterized by herniation of the cerebellum into the spinal canal. Chiari malformation type I is highly heterogeneous; therefore, an accurate explanation of the pathogenesis of the disease is often not possible. Although some studies showed the role of genetics in CMI, the involvement of genetic variations in CMI pathogenesis has not been thoroughly elucidated. Therefore, in the current study we aim to reveal CMI-associated genomic variations in familial cases.Four CMI patients and 7 unaffected healthy members of two distinct families were analyzed. A microarray analysis of the affected and unaffected individuals from two Turkish families with CMI was conducted. Analyses of single nucleotide variations (SNVs) and copy number variations (CNVs) were performed by calculation of B allele frequency (BAF) and log R ratio (LRR) values from whole genome SNV data. Two missense variations, OLFML2A (rs7874348) and SLC4A9 (rs6860077), and a 5’UTR variation of COL4A1 (rs9521687) were significantly associated with CMI. Moreover, 12 SNVs in the intronic regions of FAM155A, NR3C1, TRPC7, ASTN2, and TRAF1 were determined to be associated with CMI. The CNV analysis showed that the 11p15.4 chromosome region is inherited in one of the families. The use of familial studies to explain the molecular pathogenesis of complex diseases such as CMI is crucial. It has been suggested that variations in OLFML2A, SLC4A9, and COL4A1 play a role in CMI molecular pathogenesis. The CNV analysis of individuals in both families revealed a potential chromosomal region, 11p15.4, and risk regions that are associated with CMI.

## 1. Introduction

The Chiari malformation (CM) is a condition in which the cerebellar tonsil, which is found at the lower part of the brain right above the foramen magnum, starts extending out through the spinal canal. It blocks spinal fluid flow and can cause fluid accumulation in the canal (syringomyelia) and in the brain. Type I CM (CMI) is the most prevalent type with an incidence rate of approximately 1/1280 (Meadows et al., 2000). Chiari malformation type I can occur due to birth defects and may be detected when the patient is still an infant or during adulthood. However, in some cases—rather than a birth/genetic defect—a traumatic accident or craniocervical tumors may give rise to CMI (Merello et al., 2017). Chiari malformation type I usually comes with accompanying conditions such as syringomyelia, cerebral hydrocephalus, and basilar invagination (Shah et al., 2017). Almost 70%–80% of CMI cases display syringomyelia (Zhao et al., 2016).

The exact pathogenesis of CMI has yet to be discovered. Currently, CMI is diagnosed in clinic when there are more than 5 mm of tonsils protruding from the foramen magnum (Merello et al., 2017). The broad range of explanations for CMI does not aid in formulating a precise diagnosis. Many other conditions may be included in this type of pathogenesis; therefore, there is a need for more accurate parameters. The most classical proposed mechanism for CMI is a mesodermal defect. Neuroectodermal malformation may also be the cause of CM (Schijman, 2004).

Symptoms vary greatly among CM patients; most of the time these symptoms are as simple as a cough, headache, and nausea. Another diagnostic challenge is that decompression of the foramen magnum is handled through surgery as a way of treating CM. However, over the years risks involving the surgery have been observed; taking genetics into consideration may help in determining whether surgery is suitable (Langridge et al., 2017). Along with MRI diagnosis, genetics can be used in diagnosis. Moreover, genetics will help us to determine the genetic basis of CMI, which may elucidate its natural history or serve as a guide to treatment.

For many years, CM was considered a sporadic disease. However, family studies indicate that genetics are involved in the pathogenesis of CM. Familial aggregation shows that there could be a genetic culprit, and identifying the genes/variations responsible for the pathogenesis may aid in accurate diagnosis and treatment (Nagy et al., 2016; Merello et al., 2017).

Here we present the results of a genome-wide analysis of two Turkish families diagnosed with CMI. We present possible candidate genes associated with familial CMI pathogenesis. Moreover, chromosomal regions and CNVs that are potentially related to CMI are reported.

## 2. Materials and methods

### 2.1. Subjects

**Figure 1:**
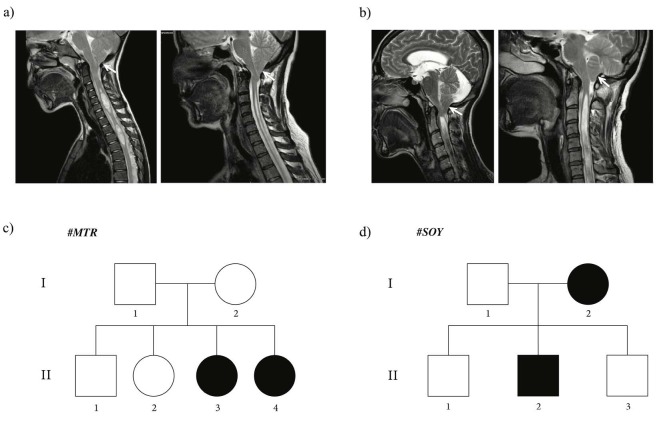
Pedigrees of two Turkish families with CMI. Squares signify males and circles signify females. Filled symbols signify affected individuals. Blood samples of every individual in the pedigrees were collected and subjected to microarray analysis. a) Pedigree of #MTR family. Individuals II-3 daughter and II-4 daughter were confirmed as affected cases. b) MR images of index patients in #MTR family. Left hand side is individual II-3, right hand side is individual II-4. c) Pedigree of #SOY family. Individuals I-2 mother and II-2 son were confirmed as affected cases. d) MR images of index patients in #SOY family. Left hand side is individual I-2, right hand side is individual II-2.

Two families with individuals diagnosed with CMI are included in the study. Families are labelled with suitable letters. The first family (# *MTR* ) consists of 6 members [2 parents (nonconsanguineous) and 4 children (1 male and 3 female)], and 2 female siblings were operated on for CMI. The second family (# *SOY* ) consists of 5 members [2 parents (nonconsanguineous) and 3 brothers], and the mother and the second son were operated on for CMI. Herniation of the tonsils through the foramen magnum, which causes brain stem pressure, is seen in Figures 1a and 1b (white arrows). Occipital headache during straining or after coughing, dysphagia, numbness in the extremities, and indifference to discrimination between hot/cold sensations, especially in the lower extremities, constituted the main clinical findings.

The other, unaffected members of both families were checked by cranial MRI, and CMI diagnoses were excluded. This study was approved by the institutional review board (2018-17/02). All procedures performed adhered to ethical guidelines. The pedigrees of both families are shown in Figure 1. In family # *MTR* , daughters II-3 and II-4 were proband individuals (Figure 1c). Daughter II-3 was operated on when she was 35 years old, and daughter II-4 was operated on when she was 37 years old. Both patients complained of occipital headaches, which were especially aggravated by coughing. In family # *SOY* , mother (I-2) and son (II-2) were the affected individuals (Figure 1d). The mother (I-2) was 40-years old when she was operated on, and her son (II-2) was 9. The mother’s complaint was untreatable headaches and her son’s was recurrent falling. The surgical procedure performed in all patients was standardized posterior fossa decompression with additional wide duraplasty.

### 2.2. Microarray analysis

The isolation of DNA from the blood samples of all individuals in both families shown in the pedigrees (Figure 1) was performed by commercially available kits, according to manufacturer recommendations (NucleoSpin DNA blood, Macherey–Nagel GmbH & Co. KG, Düren, Germany). The DNA samples were studied with an Illumina Infinium CytoSNP-850K v1.2 microarray chip and scanned on Illumina iScan platform (Illumina Inc., San Diego, CA, USA). Around 665,000 single nucleotide polymorphisms (SNPs) were genotyped for each sample. The median range of Illumina microarray chip probes is 2.53 kb. Copy number variation (CNV) analysis was performed by calculation of B allele frequency (BAF) and log R ratio (LRR) values from whole genome SNP data. Copy number variations were determined and visualized via the GenomeStudio (v.2.0.4) and cnvPartition (v.3.2.0) analysis programs provided by Illumina Inc. Human Genome Build 37 was used as a reference to align and report interested positions. The confidence threshold was 50, and GC wave correction was performed for the analysis. In order to determine loss and gain regions, the minimum probe required was set at 8, and copy number loss of heterozygosity (CNLOH) regions greater than 1 Mb were reported.

Microarray results were evaluated by two main approaches. First, SNVs between affected and unaffected members in both families were compared. Mutations in the affected members of both families were reported in Table. Second, chromosomal variations in all affected members and unaffected members were listed for both families in Supplementary Table.

**Table .** List of common single nucleotide variations in both families.

Chr. Location	Gene	Biological Process / Gene Ontologya	n	Variantb	Variant Classc	Enhanced Expressiond
9q33.3	OLFML2A	Protein homodimerization activity	1	rs7874348 (A/G)	Missense	Low tissue specificity
5q31.3	SLC4A9	Anion transmembrane transporter activity	1	rs6860077 (T/C/G)	Missense	Kidney, heart
13q.34	COL4A1	Basal membrane formation	1	rs9521687 (C/A)	5’UTR	Placenta
13q33.3	FAM155A	Calcium ion import across plasma membrane	2	rs1543002 (C/T), rs1543003 (T/A/C)	Intronic	Brain, pituitary gland
5q31.3	NR3C1	Apoptosis, cell cycle, transcription regulation	2	rs4607376 (A/G/T), rs6871464 (T/C)	Intronic	Low Tissue Specificity
5q31.1	TRPC7	Calcium transport	2	rs6893792 (T/A/C), rs10463951 (C/A/G/T)	Intronic	Adrenal gland, brain, intestine, kidney, pituitary gland, testis
9q33.1	ASTN2	Protein transport	5	rs2274414 (T/G), rs16936575 (G/A/T), rs12554069 (T/C), rs6478300 (C/A), rs10983600 (C/T)	Intronic	Low tissue specificity
9q33.2	TRAF1	Apoptosis	1	rs3761847 (G/A)	Intronic	Low tissue specificity
3p24.1	LINC00693	N/A	1	rs9875884 (T/C)	Intronic	Brain
13q33.3	MYO16	Motor activity, actin binding	4	rs76754941 (G/A), rs2759273 (T/C), rs9514937 (G/A), rs9521082 (A/G)	Intronic	Brain
13q.34	COL4A2	Basal membrane formation	7	rs73619284 (C/T), rs8001070 (G/A), rs9555689 (G/A/T), rs7317178 (C/A/T), rs12323265 (A/G), rs9515209 (C/T), rs4517640 (C/T)	Intronic	Placenta
5q31.1	FSTL4	Calcium ion binding, metal ion binding	1	rs55962865 (A/C)	Intronic	Brain
5q31.3	FGF1	Angiogenesis, differentiation	1	rs2339246 (C/G/T)	Intronic	Brain, heart muscle, kidney
5q31.3	ARHGAP26	GTPase activity	1	rs77545201 (G/T)	Intronic	Low tissue specificity
5q32	HTR4	G protein-coupled receptor activity	1	rs1820075 (C/T)	Intronic	Brain, heart muscle, intestine, pituitary gland
5q32	ADRB2	G protein-coupled receptor activity	1	rs6893517 (G/A)	Intronic	Blood
9q32	COL27A1	Extracellular matrix structural constituent	1	rs2567714 (T/C)	Intronic	Brain, uterine, cervix
9q33.1	BRINP1	Inhibits cell proliferation by negative regulation of the G1/S transition	1	rs1332453 (A/C)	Intronic	Brain
9q33.2	PHF19	Chromatin regulator	2	rs11794516 (A/G), rs2072438 (T/C)	Intronic	Low tissue specificity
9q33.2	CNTRL	Cell cycle, cell division	1	rs746182 (T/A/C)	Intronic	Low tissue specificity
9q33.3	MVB12B	Protein transport	1	rs7047946 (G/A)	Intronic	Brain
9q34.11	USP20	Endocytosis, Ubl conjugation pathway	2	rs10819567 (A/C/G), rs62583579 (C/T)	Intronic	Low tissue specificity
9q34.11	LOC101929331	N/A	2	rs10819490 (C/T), rs10988311 (T/G)	Intronic	N/A
5q23.1	LINC00992	N/A	3	rs1863977 (G/A), rs12332110 (G/A/T), rs62380137 (G/A/T)	Intronic	Pancreas, colon
5q31.1	LOC100996485	N/A	1	rs2059780 (G/A)	Intronic	N/A
5q31.3	LOC101926941	N/A	3	rs9324876 (A/G), rs959662 (A/C/G), rs9800206 (C/A/T)	Intronic	N/A
17q21.33	LOC100288866	N/A	1	rs2537720 (G/A)	Intronic	Low tissue specificity
7q22.2	LHFPL3	N/A	2	rs2470957 (G/A/T), rs12667640 (T/C)	Intronic	Brain
7q22.3	LHFPL3 - AS2	N/A	1	rs10953457 (C/T)	Intronic	Kidney
						

N/A: no known function or expression, n: number of reported variations in a gene, aAccording to Gene Cards Weizmann Institute of Science (2020). GeneCards: The Human Gene Database [online]. Website https://www.genecards.org [accessed 01 September 2020].and Gene Ontology Gene Ontology Consortium (2020). The Gene Ontology Resource [online]. Website http://geneontology.org/ [accessed 01 September 2020]. databases, bNucleotide changes were determined through dbSNP database National Center for Biotechnology Information (2020). dbSNP database [online]. Website https://www.ncbi.nlm.nih.gov/snp/ [accessed 01 September 2020]., cVariant classes were determined through Genome Browser database University of California Santa Cruz Genomics Institute (2020). Genome Browser database [online]. Website https://genome.ucsc.edu [accessed 01 September 2020., dAccording to Gene Cards database.

## 3. Results

### 3.1. Single nucleotide variations

We evaluated single nucleotide variations (SNVs) in both families and listed those that are common between the two families (Table). Most of these variations were intronic; however, there were two missense variations and one 5’UTR variation. None of the variations listed in Table were previously reported as clinically significant variations in the ClinVar database (Landrum et al., 2018).

### 3.2. Copy number variations

Genotype data of microarray results were used to perform copy number variation (CNV) analysis of the affected individuals in both families. Patient II-3 of family MTR harbored no significant CNV, whereas patient II-4 carried 3 copies of 13q13.1 and homozygote deletion of 22q13.2 variations. In family SOY, patient I-2 carried 1 copy of 7q11.21 and 11p15.4, and 3 copies of 11p12 variations, whereas patient II-2 carried 1 copy of 11p15.4 variation. In addition to the CNVs mentioned, all other nonsignificant CNVs that are common in populations and that were detected in the affected individuals of each family were listed in supplementary Table.

## 4. Discussion

Several previous studies investigated the genetic underpinnings of CMI by using genome wide analyses. One of the most comprehensive studies of CMI included 23 families with 71 affected individuals and microarray data with 10K SNP Chip analysis. Researchers reported a significant linkage on chromosomes 9 and 15 (Boyles et al., 2006). In another comprehensive study, Markunas et al. conducted the largest genome linkage screen in 66 families and 367 individuals. They reported a maximum logarithm of distance (LOD) score of 3 for chromosomes 8 and 12 and listed candidate associated genes such as SUZ12, NF1, and LR6 (Markunas et al., 2013). A similar exploratory analysis of copy number variations in a Turkish family with 2 members affected by CMI failed to identify any significant CNV or gene candidate (Keser et al., 2019). Various studies reported different results. Therefore, we aim to report potential candidate CNVs and genes for familial CMI.

**Figure 2:**
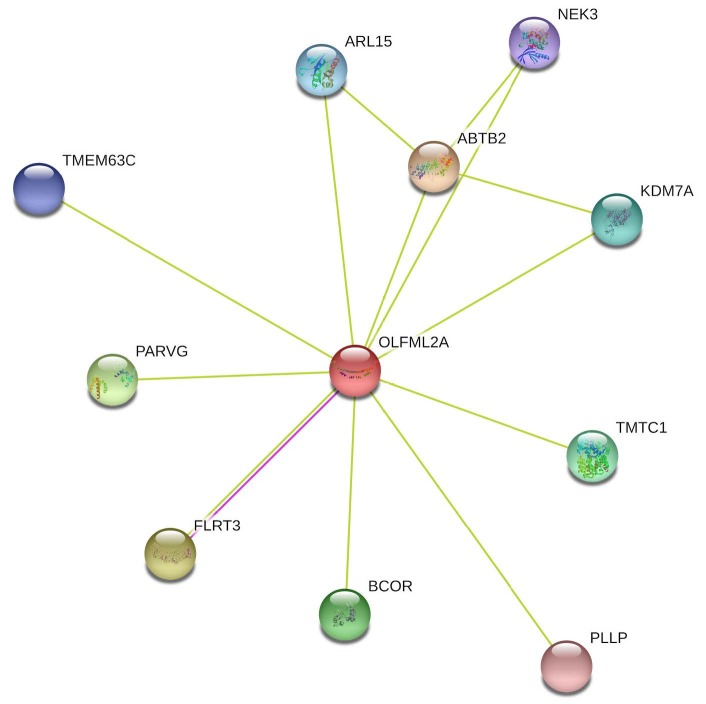
STRING functional protein association network of OLFML2A gene (accessed 01 September 2020).

A missense variation was detected in *OLFML2A* gene (rs7874348) (Table). An SNV was found at exon 7 of the gene and caused Thr to Ala amino acid conversion. *OLFML2A* (olfactomedin-like 2A) is an olfactomedin-domain–containing protein, and its function in a cell has not been extensively explored (Sistani et al., 2013). Previous studies state that *OLFML2A* gene has a role in extracellular matrix (ECM) modeling (Furutani et al., 2005; Hong et al., 2019). Since our knowledge about the function of *OLFML2A* gene is limited, we used the STRING database to determine the functional protein association network for our gene of interest (Szklarczyk et al., 2015). Networks shown in Figure 2 demonstrate functional proteins that are connected with *OLFML2A.* Some of the genes shown in the network might be helpful for understanding the cellular processes involving *OLFML2A* (Figure 2). *NEK3* gene encodes for a serine/threonine protein kinase which has a role in neuronal morphogenesis through microtubule acetylation (Chang et al., 2009). *FLRT3* gene was found to be expressed during craniofacial development (Gong et al., 2009), and it functions in cell-cell adhesion and migration during cortical folding (Lu et al., 2015; Del Toro et al., 2017). Lastly, *KDM7A* gene in the network encodes for the histone–demethylase required for brain and craniofacial development (Qi et al., 2010; Tsukada et al., 2010). Hence, we propose that *OLFML2A* gene may be an essential gene in the pathogenesis of CMI due to its role in development of brain structures.

Another missense variation was detected in *SLC4A9* gene (rs6860077) and, similarly, this variation has not been previously reported. It is found at exon 12 of the gene and causes Ile to Met amino acid conversion. Solute carrier family 4 member 9 ( *SLC4A9* ) belongs to the *SCL4A* family of anion exchangers. Peña-Münzenmayer et al. reported that *SLC4A9* gene has a role in fluid secretion regulation and is specifically responsible for Cl- exchange in the cell (Peña-Münzenmayer et al., 2016). It has been shown that cerebrospinal fluid (CSF) secretion is controlled by Na+, Cl- , and HCO3- transportation (Brown et al., 2004). Disrupted CSF flow in the brain may contribute to many pathologies, including CMI, in which the flow is further disrupted due to cerebellar herniation (Buell et al., 2015).

Another significant SNV (rs9521687) was detected in *COL4A1* gene as a 5’UTR variation. This particular SNV has not been associated with any condition, and there are no published studies related to rs9521687. Type IV collagens can only be found in basement membranes, and the family consists of 6 distinct a-chains. The earliest discovered chains, a1(IV) and a2(IV), are considered classical chains, and they are encoded by *COL4A1* and *COL4A2* , respectively. Classical chains are expressed both during development and adulthood; however, four other chains are only expressed during development. The a1a1a2 triple helix is formed and secreted to the extracellular matrix (ECM), which then becomes a molecular scaffold to other ECM components (Khoshnoodi et al., 2008). Type IV collagen family variations have been associated with various sporadic and genetic diseases. *COL4A1* gene variations may cause poor speech development, cerebral palsy, epilepsy (Pasternak et al., 1980), porencephaly and adult stroke (Gould et al., 2006; van der Knaap et al., 2006), and encephalopathy (Yaramis et al., 2020). These diseases all suggest that *COL4A1* mutations may cause impaired vasculature formation in the brain. Urbizu et al. stated that variations affecting fetal vascular development might cause susceptibility to CMI (Urbizu et al., 2013).

Five of the significantly associated variations (rs1543002, rs10463951, rs4607376, rs6478300, and rs3761847) were reported previously. The SNV (rs1543002) in family with sequence similarity 155 member A gene ( *FAM155A* ) was reported as a significant SNV for patients diagnosed with bipolar disease and attempted suicide (Willour et al., 2012). Although there is not much information about the function of *FAM155* family genes in humans, studies showed that it is associated with calcium channel activities (Gaudet et al., 2011). Calcium channels are known to have a role in neural tube closure during developmental stages (Abdul-Wajid et al., 2015), and CMI can be classified as an neural tube defect (NTD) in some cases. The SNV (rs4607376) in nuclear receptor subfamily 3 group C member 1 gene ( *NR3C1* ), which is a glucocorticoid receptor, was previously reported in only one clinical study and it was related with systemic lupus erythematosus (Chen et al., 2017). Although no previous publications have established a relationship between autoimmune diseases and CMI, there have been cases reported suggesting that autoimmune diseases and CM pathogenesis might be related (Ortolani, 2014).

The 3 remaining intronic SNVs (rs10463951) in transient receptor potential cation channel subfamily C member 7 gene ( *TRPC7* ), (rs6478300) (Wang et al., 2009) in astrotactin 2 gene ( *ASTN2* ), and (rs3761847) (Huang et al., 2019; Plenge et al., 2007) in TNF receptor associated factor 1 gene ( *TRAF1* ) are all reportedly related to the autoimmune disease rheumatoid arthritis (RA). *TRPC7* gene is a calcium channel that has a role mainly in keratinocyte development (Beck et al., 2006). *ASTN2* gene encodes for a protein which regulates surface proteins by endocytic trafficking and protein degradation (Behesti et al., 2018). *ASTN2* is reported to form a complex with *ASTN1* which plays a role in neuronal migration, and both genes are highly expressed in the cerebellum (Wilson et al., 2010). *TRAF1* was first identified as a part of the TNFR2 signaling complex, and it also has relationships with other TNFR superfamily members that play a role in prosurvival signaling. *TRAF1* is a well-studied gene, and it has been associated with many different diseases such as B-cell–related cancers, RA, cardiovascular diseases, and many infectious diseases (Edilova et al., 2018).

The CNV analysis provided us with a list of candidate chromosomal regions that are potentially associated with CMI. Furthermore, 11p15.4 one-copy deletion is inherited via mother to son in family SOY.

## 5. Conclusion

Elucidation of the molecular etiology of CMI by using familial cases is essential for such a complex disease. We conducted molecular karyotyping and SNV analysis for two families and listed important single nucleotide variations with regard to their mutation status and possible roles in CMI etiology. We propose that mutations in OLFML2A, SLC4A9, and COL4A1 genes may have a role in the familial form of CMI. Although a number of chromosomal regions were listed as potential regions associated with CMI, only 11p15.4 chromosome regions were CNVs that were inherited by a son via an affected mother. Our explorative results should be confirmed through further familial CMI case studies and molecular studies.

## Informed consent

This study was approved by the ethical committee of Bahçeşehir University, School of Medicine (BAU2018-17/02). All patients and family members provided informed consent. All procedures were performed according to the relevant ethical standards.

Supplementary Materials
