# Supplementary material for: Genome-wide identification of Chiari malformation type I associated candidate genes and chromosomal variations
Source: Turk J Biol. 2020 Dec 14;44(6):449–56. doi: 10.3906/biy-2009-19 (PMC7759189; doi:10.3906/biy-2009-19)
Supplement: Supplementary file 1 — Supplementary Materials [file turkjbio-44-449-sup001.pdf]

Supplementary data

Supplementary Table 1. Chromosomal variations in MTR family.

Index	Variation	Copy Number	Chromosome	Start	End	Lenght	Probe	Region
II-3	LOF	0	19	41381495	41381648	154	14	q13.2
II-3	LOF	0	19	41385334	41385869	536	12	q13.2
II-3	LOF	0	19	41386421	41386814	394	12	q13.2
II-3	LOF	0	22	24301392	24301824	433	15	q11.23
II-3	LOF	0	22	24302485	24302603	119	6	q11.23
II-3	LOF	0	22	42522134	42522313	180	7	q13.2
II-3	LOH	2	1	6145365	7613598	1468234	394	p36.31; p36.23
II-3	LOH	2	1	70257972	71841054	1583083	225	p31.1
II-3	LOH	2	1	93396819	94462243	1065425	187	p22.1
II-3	LOH	2	1	163508344	165825989	2317646	546	q23.3; q24.1
II-3	LOH	2	2	17822	1833668	1815847	495	p25.3
II-3	LOH	2	2	131112197	133040424	1928228	159	q21.1; q21.2
II-3	LOH	2	2	186224432	189857611	3633180	486	q32.1; q32.2
II-3	LOH	2	2	189859032	218663979	28804948	5881	q32.2; q32.3; q33.1; q33.2; q33.3; q34; q35
II-3	LOH	2	3	101232093	109465442	8233350	1462	q12.3; q13.11; q13.12; q13.13
II-3	LOH	2	3	186551711	188766239	2214529	705	q27.3; q28
II-3	LOH	2	5	17199259	27707936	10508678	1766	p15.1; p14.3; p14.2; p14.1
II-3	LOH	2	5	61758546	68829459	7070914	1181	q12.1; q12.2; q12.3; q13.1; q13.2
II-3	LOH	2	5	70398445	72911803	2513359	416	q13.2
II-3	LOH	2	7	152584806	159124173	6539368	2092	q36.1; q36.2; q36.3
II-3	LOH	2	13	106582808	115106996	8524189	2635	q33.2; q33.3; q34
II-3	LOH	2	14	38996493	40235485	1238993	179	q21.1
II-3	LOH	2	14	59214061	62979707	3765647	723	q23.1; q23.2
II-3	LOH	2	14	94579327	95678141	1098815	427	q32.12; q32.13
II-3	LOH	2	14	95684801	107283150	11598350	2811	q32.13; q32.2; q32.31; q32.32; q32.33
II-3	LOH	2	15	24008242	25907528	1899287	406	q11.2; q12
II-3	LOH	2	15	66372477	77276650	10904174	2552	q22.31; q22.32; q22.33; q23; q24.1; q24.2; q24.3
II-3	LOH	2	16	2133716	5180366	3046651	906	p13.3
II-3	LOH	2	16	22817446	27061619	4244174	1308	p12.2; p12.1
II-3	LOH	2	17	15647723	17200920	1553198	305	p12; p11.2
II-3	LOH	2	22	47105302	51214796	4109495	1515	q13.31; q13.32; q13.33
II-3	LOH	2	X	41302842	54735349	13432508	1963	p11.4; p11.3; p11.23; p11.22
II-3	LOH	2	X	107253323	108530481	1277159	126	q22.3
II-3	LOH	2	X	153363118	154913173	1550056	441	q28
II-3	LOH	2	X	88484868	89798620	1313753	193	q21.31
**II-4**	GOF	3	1	110230206	110241385	11180	72	p13.3
**II-4**	GOF	3	13	32914933	32915231	299	87	q13.1
**II-4**	LOF	0	19	41349596	41349656	61	9	q13.2
**II-4**	LOF	0	19	41381553	41381648	96	3	q13.2

Supplementary Table 1. Continued.

Index	Variation	Copy Number	Chromosome	Start	End	Lenght	Probe	Region
**II-4**	GOF	3	22	24374253	24385622	11370	119	q11.23
**II-4**	LOF	0	22	42522134	42522313	180	7	q13.2
**II-4**	LOH	2	1	6145365	7613598	1468234	394	p36.31; p36.23
**II-4**	LOH	2	1	163508344	165825989	2317646	546	q23.3; q24.1
**II-4**	LOH	2	1	190520424	191922512	1402089	196	q31.1; q31.2
**II-4**	LOH	2	2	4247903	7256134	3008232	731	p25.3; p25.2; p25.1
**II-4**	LOH	2	3	22204135	24882331	2678197	626	p24.3; p24.2
**II-4**	LOH	2	3	84961960	86378386	1416427	198	p12.1
**II-4**	LOH	2	3	101232093	122707286	21475194	4080	q12.3; q13.11; q13.12; q13.13; q13.2; q13.31; q13.32; q13.33; q21.1
**II-4**	LOH	2	4	38380620	40684941	2304322	569	p14
**II-4**	LOH	2	4	40686808	49620838	8934031	1556	p14; p13; p12; p11
**II-4**	LOH	2	4	52684820	55432159	2747340	467	q11; q12
**II-4**	LOH	2	4	180961974	182534983	1573010	482	q34.3
**II-4**	LOH	2	7	1604320	2911972	1307653	384	p22.3; p22.2
**II-4**	LOH	2	7	39423916	40485935	1062020	140	p14.1
**II-4**	LOH	2	8	172972	10464604	10291633	3729	p23.3; p23.2; p23.1
**II-4**	LOH	2	8	10480500	13002472	2521973	647	p23.1; p22
**II-4**	LOH	2	8	14020120	15045555	1025436	309	p22
**II-4**	LOH	2	8	23194680	43791691	20597012	4094	p21.3; p21.2; p21.1; p12; p11.23; p11.22 p11.21; p11.1
**II-4**	LOH	2	8	46936719	54991386	8054668	1202	q11.1; q11.21; q11.22; q11.23
**II-4**	LOH	2	9	25581774	45755225	20173452	2904	p21.3; p21.2; p21.1; p13.3; p13.2; p13.1; p12; p11.2
**II-4**	LOH	2	9	70906647	81587711	10681065	2205	q21.11; q21.12; q21.13; q21.2; q21.31
**II-4**	LOH	2	10	32054404	33708671	1654268	247	p11.22
**II-4**	LOH	2	11	84837883	86448092	1610210	272	q14.1; q14.2
**II-4**	LOH	2	13	106582808	111438085	4855278	1584	q33.2; q33.3; q34
**II-4**	LOH	2	15	24008242	25907528	1899287	406	q11.2; q12
**II-4**	LOH	2	16	24326722	27061619	2734898	689	p12.1
**II-4**	LOH	2	18	37617494	42358137	4740644	789	q12.3
**II-4**	LOH	2	20	1916836	4595025	2678190	754	p13
**II-4**	LOH	2	21	36528182	37981249	1453068	357	q22.12; q22.13
**II-4**	LOH	2	22	47105302	51214796	4109495	1515	q13.31; q13.32; q13.33
**II-4**	LOH	2	X	153363118	154913173	1550056	441	q28

Supplementary Table 2. Chromosomal variations in SOY family.

Index	Variation	Copy Number	Chromosome	Start	End	Lenght	Probe	Region
I-2	GOF	3	1	110220086	110245765	25680	85	p13.3
I-2	LOF	1	7	64693037	65087974	394938	43	q11.21
I-2	LOF	1	11	5248193	5248250	58	25	p15.4
I-2	GOF	3	11	37582575	38114401	531827	98	p12
I-2	LOF	0	19	41385334	41385775	442	9	q13.2
I-2	LOF	0	19	41386421	41386677	257	9	q13.2
I-2	LOF	0	22	42522134	42522313	180	7	q13.2
I-2	LOH	2	1	71051569	73583976	2532408	334	p31.1
I-2	LOH	2	3	28984225	50013778	21029554	5513	p24.1; p23; p22.3; p22.2; p22.1; p21.33; p21.32; p21.31
I-2	LOH	2	3	50129399	53662306	3532908	568	p21.31; p21.2; p21.1
I-2	LOH	2	4	8924210	10061147	1136938	155	p16.1
I-2	LOH	2	4	153882282	154995463	1113182	278	q31.3
I-2	LOH	2	5	63221842	64265857	1044016	131	q12.3
I-2	LOH	2	6	27339418	28641735	1302318	341	p22.1
I-2	LOH	2	7	86443168	87754969	1311802	373	q21.12
I-2	LOH	2	9	135425830	141068637	5642808	2342	q34.13 q34.2; q34.3
I-2	LOH	2	12	116775148	118395127	1619980	467	q24.21; q24.22; q24.23
I-2	LOH	2	13	111294811	112868642	1573832	492	q34
I-2	LOH	2	14	52006390	54290388	2283999	496	q22.1; q22.2
I-2	LOH	2	14	55997611	65701286	9703676	2108	q22.3; q23.1; q23.2; q23.3
I-2	LOH	2	15	43818532	45440621	1622090	300	q15.3; q21.1
I-2	LOH	2	16	3370894	6896128	3525235	1231	p13.3
I-2	LOH	2	17	13671552	22234751	8563200	1690	p12 p11.2; p11.1
I-2	LOH	2	17	25311244	31320695	6009452	1060	q11.1; q11.2
I-2	LOH	2	19	41928183	59097933	17169751	5017	q13.2; q13.31; q13.32; q13.33; q13.41; q13.42; q13.43
I-2	LOH	2	21	25902463	39947747	14045285	3245	q21.2; q21.3; q22.11; q22.12; q22.13; q22.2
I-2	LOH	2	22	31535872	32662384	1126513	185	q12.2; q12.3
I-2	LOH	2	22	45395342	49074301	3678960	1247	q13.31; q13.32
I-2	LOH	2	22	49591992	51214796	1622805	626	q13.33
I-2	LOH	2	X	86683205	87698139	1014935	225	q21.31
**II-2**	GOF	3	1	110228436	110245765	17330	84	p13.3
**II-2**	LOF	1	11	5248193	5248250	58	25	p15.4
**II-2**	LOF	1	16	30208520	30212911	4392	29	p11.2
**II-2**	LOF	1	19	11224269	11224319	51	16	p13.2
**II-2**	LOF	1	19	11231099	11231159	61	23	p13.2
**II-2**	LOF	1	19	41381495	41381648	154	14	q13.2
**II-2**	LOF	0	19	41382010	41382256	247	3	q13.2
**II-2**	LOF	1	19	41384103	41385869	1767	39	q13.2
**II-2**	LOF	0	22	24301392	24301695	304	12	q11.23
**II-2**	LOF	1	22	24301824	24302487	664	24	q11.23

Supplementary Table 2. Continued.

Index	Variation	Copy Number	Chromosome	Start	End	Lenght	Probe	Region
**II-2**	GOF	3	22	24375632	24385472	9841	102	q11.23
**II-2**	LOF	0	22	42522134	42522397	264	10	q13.2
**II-2**	GOF	2	X	48303837	48386632	82796	39	p11.23
**II-2**	GOF	2	X	99661925	99663226	1302	31	q22.1
**II-2**	LOF	0	Y	2654333	10068588	7414256	1564	p11.31; p11.2
**II-2**	LOF	0	Y	13133499	19567718	6434220	2157	q11.1; q11.21; q11.221
**II-2**	LOF	0	Y	20804835	24522333	3717499	1197	q11.222; q11.223
**II-2**	LOF	0	Y	28509482	28817636	308155	32	q11.23; q12
**II-2**	LOH	2	1	54124121	68741305	14617185	3487	p32.3; p32.2; p32.1; p31.3
**II-2**	LOH	2	1	118941488	120405606	1464119	290	p12
**II-2**	LOH	2	2	40078780	47639559	7560780	2169	p22.1; p21
**II-2**	LOH	2	2	47639598	49190917	1551320	1207	p21; p16.3
**II-2**	LOH	2	2	49194702	51819167	2624466	582	p16.3
**II-2**	LOH	2	3	417207	3518222	3101016	1023	p26.3; p26.2
**II-2**	LOH	2	3	3526289	16281960	12755672	4051	p26.2; p26.1; p25.3; p25.2; p25.1
**II-2**	LOH	2	3	53599972	60113719	6513748	1523	p21.1; p14.3; p14.2
**II-2**	LOH	2	4	8924210	10416360	1492151	241	p16.1
**II-2**	LOH	2	4	76475804	84664343	8188540	1570	q21.1; q21.21; q21.22; q21.23
**II-2**	LOH	2	4	121814232	124359046	2544815	504	q27; q28.1
**II-2**	LOH	2	4	154938646	163486514	8547869	1766	q31.3; q32.1; q32.2
**II-2**	LOH	2	4	174490092	176125249	1635158	366	q34.1
**II-2**	LOH	2	5	88215821	90698051	2482231	478	q14.3
**II-2**	LOH	2	6	162699	6780870	6618172	1943	p25.3; p25.2; p25.1
**II-2**	LOH	2	6	27339418	28470909	1131492	303	p22.1
**II-2**	LOH	2	7	134976271	151415504	16439234	4120	q33; q34; q35; q36.1
**II-2**	LOH	2	8	73231464	92409428	19177965	3207	q13.3; q21.11; q21.12; q21.13; q21.2; q21.3
**II-2**	LOH	2	8	92419127	112996602	20577476	3869	q21.3; q22.1; q22.2; q22.3; q23.1; q23.2; q23.3
**II-2**	LOH	2	9	4355828	7231323	2875496	751	p24.2; p24.1
**II-2**	LOH	2	9	87666681	94821398	7154718	1568	q21.33; q22.1; q22.2; q22.31
**II-2**	LOH	2	9	95700701	105041325	9340625	1947	q22.31; q22.32; q22.33; q31.1
**II-2**	LOH	2	9	135284962	136411084	1126123	582	q34.13; q34.2
**II-2**	LOH	2	9	136414531	140377226	3962696	1654	q34.2; q34.3
**II-2**	LOH	2	10	82704505	90740501	8035997	1817	q23.1; q23.2; q23.31
**II-2**	LOH	2	10	105721994	117935672	12213679	2482	q24.33; q25.1; q25.2; q25.3
**II-2**	LOH	2	10	118201081	120767115	2566035	680	q25.3; q26.11
**II-2**	LOH	2	11	71287843	84543955	13256113	2912	q13.4; q13.5; q14.1
**II-2**	LOH	2	11	84545984	85993211	1447228	219	q14.1; q14.2
**II-2**	LOH	2	11	86040620	94708891	8668272	1742	q14.2; q14.3; q21
**II-2**	LOH	2	11	114074756	120678788	6604033	1893	q23.2; q23.3
**II-2**	LOH	2	12	74028301	75054053	1025753	124	q21.1

Supplementary Table 2. Continued.

Index	Variation	Copy Number	Chromosome	Start	End	Lenght	Probe	Region
**II-2**	LOH	2	12	106729425	118395127	11665703	2819	q23.3; q24.11; q24.12; q24.13; q24.21; q24.22; q24.23
**II-2**	LOH	2	12	129107131	132104045	2996915	1127	q24.32; q24.33
**II-2**	LOH	2	12	132124118	133801770	1677653	437	q24.33
**II-2**	LOH	2	15	40725601	41829178	1103578	208	q15.1
**II-2**	LOH	2	15	98720899	100928615	2207717	732	q26.3
**II-2**	LOH	2	16	92688	4417797	4325110	1683	p13.3
**II-2**	LOH	2	16	84314573	85915218	1600646	657	q24.1
**II-2**	LOH	2	17	26800204	27889643	1089440	196	q11.2
**II-2**	LOH	2	18	30089478	43049523	12960046	2186	q12.1; q12.2; q12.3
**II-2**	LOH	2	18	43065107	44181227	1116121	352	q12.3; q21.1
**II-2**	LOH	2	18	55839162	59015339	3176178	832	q21.31; q21.32; q21.33
**II-2**	LOH	2	18	59020751	70724185	11703435	2705	q21.33; q22.1; q22.2; q22.3
**II-2**	LOH	2	20	19885390	26266524	6381135	1189	p11.23; p11.22; p11.21; p11.1
**II-2**	LOH	2	20	29459599	36296688	6837090	1092	q11.21; q11.22; q11.23
**II-2**	LOH	2	20	36305516	39043958	2738443	525	q11.23; q12
**II-2**	LOH	2	20	39045734	40070246	1024513	238	q12
**II-2**	LOH	2	20	40078085	55450089	15372005	3982	q12; q13.11; q13.12; q13.13; q13.2; q13.31
**II-2**	LOH	2	21	18457828	21038473	2580646	613	q21.1
**II-2**	LOH	2	21	25902463	36223627	10321165	2267	q21.2; q21.3; q22.11; q22.12
**II-2**	LOH	2	X	519500	1846919	1327420	228	p22.33

GOF: gain of function, LOH: loss of heterozygosity, LOF: loss of function.
